# Survival and predictors among preterm neonates admitted at University of Gondar comprehensive specialized hospital neonatal intensive care unit, Northwest Ethiopia

**DOI:** 10.1186/s13052-018-0597-3

**Published:** 2019-01-07

**Authors:** Ayenew Engida Yismaw, Abebaw Addis Gelagay, Malede Mequanent Sisay

**Affiliations:** 1 School of midwifery, College of Medicine and Health Science, University of Gondar, Gondar, Ethiopia; 2 Department of Reproductive Health, Institute of Public Health, College of Medicine and Health Science, University of Gondar, Gondar, Ethiopia; 3 Department of Epidemiology and Biostatistics, Institute of Public Health, College of Medicine and Health Science, University of Gondar, Gondar, Ethiopia

**Keywords:** Ethiopia, Preterm neonate, Time to death, Neonatal intensive care unit

## Abstract

**Background:**

Prematurity accounts about 1 million neonatal deaths worldwide and the second causes of both neonatal and under five-child mortality. Neonatal mortality accounts for 43% of under-five child mortality in Ethiopia. From this preterm is the second leading cause of death and is steadily increased in low-income countries. Therefore, the aim of this study was to assess time to death and predictors among preterm neonates admitted in University of Gondar comprehensive specialized hospital neonatal intensive care unit North West Ethiopia 2018.

**Methods:**

Institution-based retrospective follow-up study was conducted among 516 preterm neonates from January 2016 to March 2018. Data were extracted retrospectively from patients’ records using a pretested structured checklist. Descriptive summary statistics like median survival time, Kaplan Meier failure estimation curve and Log-rank test were computed. Bivariate and multivariable Gompertz parametric hazards models were fitted to identify the predictors of mortality. Hazard ratio with a 95% confidence interval was calculated and *p*-values < 0.05 were considered statistically significant.

**Results:**

The proportion of preterm neonatal death in this study was 28.8% (95%CI (25.1, 32.9)). Home delivery (AHR = 2.25, 95% CI (1.03, 4.88)), hyaline membrane disease (AHR =3.21, 95% CI (1.96, 5.25)), gestational age, (AHR = 0.82, 95% CI (0.74, 0.91)), cry immediately at birth (AHR = 1.74, 95% CI (1.19, 2.53)), kangaroo mother care (AHR = 0.24, 95%CI (0.11, 0.52)), presence of jaundice (AHR = 1.62, 95%CI (1.12, 2.54)) and hypoglycemia at admission (AHR = 1.75, 95%CI (1.21, 2.54)) were found to be significant predictors of time to death for preterm neonates.

**Conclusion:**

Proportion of preterm neonatal death in this study was high. Home delivery, Jaundice, hypoglycemia, gestational age, cry immediately at birth, kangaroo mother care and hyaline membrane disease were significant predictors of time to death.

## Introduction

Approximately 3.1 million and 2.9 million neonatal deaths were reported in 2010 and 2014, respectively worldwide accounting for 40% of the under 5 mortality. Despite a steady decline, neonatal mortality is not satisfactory in African countries [1, 2]. Globally, almost 3 in 4 neonatal deaths were caused by preterm birth complications within the first week after birth, accounting for 35% of all neonatal deaths [3]. Worldwide, neonatal mortality has been reported to be caused by infection (36%), preterm birth (28%) and birth asphyxia (23%) [4–6].

Preterm (PT), a birth before 37 completed weeks of gestation, is the most frequent cause of neonatal death and the second leading cause of both neonatal and under-five mortality and most frequent cause of multiple short- and long-term health threats worldwide. The risk of the problem is 12 times higher in African [7]. Lack of full immunologic competence places preterm infants at increased risk for multiple infectious processes leading to long-term sequelae of prematurity like neurodevelopmental disorders and chronic lung disease [8].

The Ethiopian Demographic and Health Surveys reported that neonatal deaths increased from 32% in 2005 to 43% in the 2016 and according to report of United Nations of children fund, in Ethiopia preterm birth which accounts 23% was believed to be a major and direct cause of neonatal death [9, 10]. Causal factors linked to preterm birth include medical conditions of the mother or fetus, genetic influences, environmental exposure, infertility treatments, behavioral and socio-economic factors, medically indicated preterm deliveries as well as iatrogenic prematurity [11, 12].

Studies in the world on progress, priorities, and survival of the worlds’ newborns and children reported that preterm neonatal mortality ranged from 15 to 36, percent [1]. Findings from the worlds’ low to middle-income countries on the incidence, risk factors and causes of neonatal mortality showed that 34–40% were contributed by preterm [5]. Hospital-based studies in Africa on the burden and predictors of neonatal mortality reported that preterm was account about 15.7 to 29.6% Studies in Ethiopia on causes, survival, predictors, and implications of preterm neonatal mortality reported different findings ranged from 18% up to more than 40%.

Different studies conducted so far in the different area reported risk factors for preterm neonatal death: Being rural residency, maternal age less than 20 and greater than 35 [5, 13], place of birth.

Obstetric risk factors for preterm neonatal death were not having ANC, being prim para [4], having any pregnancy complications, Labour and delivery complications, having previous bad obstetric history, Being multiple pregnancies.

Neonatal related risk factors for preterm neonatal survival were being male sex (), having low birth weight at birth, gestational age (GA) at birth and neonatal congenital malformations, presence of neonatal clinical problems such as, respiratory distress syndrome (RDS), perinatal asphyxia (PNA), hyaline membrane disease (HMD), jaundice, hypoglycemia, hypothermia and neonatal sepsis [6, 13], timely initiation of breastfeeding upon birth and kangaroo mother care (KMC) were reported as factors of preterm neonatal death.

Even if premature birth is not an acute disease, it is one of the major causes of infants’ death and it continues to be significant public health problem by increasing the average cost of medical care for a premature and low birth-weight baby for the first year of life for developing country like Ethiopia. These high medical expenses could burden both the parents, families as well as the community. Therefore, this is the dual agenda to prevent preterm birth and address the survival gap of premature babies which requires a comprehensive research strategy to end the preventable deaths of newborns and under-five children.

## Methodology

### Study design and setting

Institution-based retrospective follow-up study was conducted among preterm neonates admitted in NICU from January 2016 to March 2018 to estimate the time to death and predictors of preterm neonates admitted in University of Gondar comprehensive specialized hospital neonatal intensive care unit. University of Gondar specialized referral hospital is one of the largest teaching hospital found in Amhara region providing tertiary level care for more than seven million people in the North West part of the country coming from Amhara Tigray and Benishangul Gumuz regions. It is located at 727 km and 180 km far from Addis Ababa and Bahir Dar, the capital city of Ethiopia and Amhara region to Northwest respectively. The hospital serves as both comprehensive and referral level care. Neonatology is a unit under pediatrics and child health department. The department has 1:5 nurse-patient and 1:10 physician-patient ratio for 24 h and seven days with total of 40 neonatal beds. It provides an outpatient and inpatient medical service for neonates with free of charge. Neonatal intensive care unit particularly offers intensive care for neonates in an inpatient basis by providing procedures like nCPAP, phototherapy, mechanical ventilation and exchange transfusion.

### Sample size and sampling procedure

All preterm neonates who were admitted to neonatal intensive care unit at the University of Gondar comprehensive specialized hospital. Thus, preterm neonates admitted to NICU with a gestational age of less than 37 completed weeks were source population and all preterm neonates who were admitted to neonatal intensive care unit (NICU) at University of Gondar comprehensive specialized hospital from January 2016 to March 2018 were the study population. The sample size was determined as follow by the following formula:

E = ((Zα/2 + Zβ)^2)/((ln(HR))^2 q1q0) Then the sample size *N* = E/d with the assumptions of; number of event (E), probability of the event (d), Where q1 was probability of event in previous study and q0 = 1- q1 and values of Z α/2 and Zβ as 1.96& 0.842 at 95% confidence level respectively. Taking 34.9% proportion of death of preterm neonates. But we took all preterm neonates admitted in NICU and easily accessed their registrations during the study period which were 516.

### Operational definition

In this study, perinatal asphyxia is defined as profound metabolic or mixed acidemia, persistence of an Apgar score of 0–3 for longer than 5 min, neonatal neurologic sequelae (e.g. seizures, coma, hypotonia, and inability to suck/cry).

In this study, clinical sepsis is defined as Clinical sign symptoms with presence of risk factors, lab tests (microscopic) or confirmed by blood culture.

### Data collection method and instruments

Prior to data collection, the records were reviewed and preterm neonatal cards were identified by their medical registration/card number. Then data were extracted using structured and pretested data extraction checklist which was prepared in English from HMIS registration format and patient’s card. Trained midwife professionals had collected the data and the patient card clerks supported them by identifying the cards of preterm neonates.

### Data quality assurance and analysis

The data were entered into EPI info version 7 after checking for completeness and imported to Stata version 14 statistical software for further analysis. Exploratory and statistical data analysis of survival time, Kaplan Meier failure estimation curve and Log-rank test were done. Proportional hazard assumption was checked both graphically and hypothesis test called Shenfield residual test. At the same time, the Schoenfeld residual test was done for almost all variables and met the proportional hazard assumption. Schoenfeld residuals test (global test) showed that PHA was satisfied.

After the proportional hazard assumption checked; by comparing models, a more parsimonious hazard model was chosen by means of the log likelihood ratio (LR) test and the Akaike Information Criterion (AIC). Under the parametric approach, the baseline hazard is defined as a parametric function and the vector of its parameters are estimated together with the regression coefficients. Thus in the case of a comparison between models, we compared using both log-likelihood ratio test and AIC value. The best-fitted model was chosen using AIC and those having the smallest AIC were considered as a best-fitted model. Then, parametric models were done for time to death of preterm neonates to identify the potential determinants. The hazard ratio is used as a measure of the probability of death, assuming that the Survival model is usually expressed in terms of hazard function.

Similarly, the goodness of model fitness also checked using the Cox-Snell residual test.

## Results

### Socio-demographic and obstetric characteristics of mothers

In this study, 516 neonatal medical charts were included in the analysis. Two third (66.86%) of the mothers were resided out of Gondar town and 30(5.8%) of them had no ANC visit during the index pregnancy. About one fifth 417(80.81%) of mothers were in the age range of 20–34 years and their mean age was 26.52 with 95%CI (26.03, 27.00) years. About 90 (17.44%) of the participants were grand multipara (5 or more) which is a known risk factor for both maternal and perinatal death. Twenty-three (4.5%) of the participants was delivered at home which is the major cause of maternal and neonatal death. **(**Table 1**).**

**Table 1:** Socio-demographic and obstetric characteristics of mothers of preterm neonates admitted in NICU at University of Gondar comprehensive specialized Hospital from January 2016 to March 2018 (*n* = 516)

Characteristics	Frequency	Percent
Maternal residence		
Gondar town	171	33.14
Out of Gondar town	345	66.86
Age of women in years		
< 20	41	7.95
20–34	417	80.81
> =35	58	11.24
Had ANC in index pregnancy		
Yes	486	94.2
No	30	5.8
Parity (Number of Births)		
I	230	44.57
II-IV	196	37.99
≥ V	90	17.44
Complication during index pregnancy		
Yes	120	23.26
No	396	76.74
Previous bad obstetrics history		
Yes	80	15.5
No	436	84.5
Type of pregnancy		
Singleton	331	64.15
Multiple	185	35.85
Onset of labor		
Elective caesarean section	56	10.85
Spontaneous	425	82.37
Induced	35	6.78
Place of birth		
Home	23	4.50
Health center	120	23.25
Hospital	373	72.25
Mode of delivery		
Spontaneous vaginal delivery	384	74.42
Caesarean section	117	22.67
Instrument assisted delivery	15	2.91
Duration of labour in hours (*n* = 460)		
< 4	52	11.30
4–18	354	77.00
> 18	54	11.70

### Characteristics of the preterm neonates

Among 516 preterm neonates, 303 (58.73%) were males and 109 (21.12%) were small for gestational age. About one-sixth 82 (15.89%) had a body temperature of greater or equal to 360C measured within 1 h of admission and 371 (71.9%) were heated under radiant warmer. **(**Table 2).

**Table 2:** Characteristics of preterm neonates admitted in NICU at University of Gondar comprehensive specialized Hospital from January 2016 to March 2018 (*n* = 516)

Characteristics	Frequency	Percent
Sex of the neonate		
Male	303	58.73
Female	213	41.27
Gestational age		
< 32 weeks	107	20.74
32–35 weeks	269	52.13
35–37 weeks	140	27.13
Weight for gestational age at birth		
Small	109	21.12
Appropriate	407	78.88
Newborn cry immediately at birth		
Yes	385	74.61
No	131	25.39
Bag and mask resuscitation at birth		
Yes	209	40.5
No	307	59.5
Newborns temperature with in 1 h of admission		
< =32	15	2.91
32.1–34	151	29.26
34.1–35	158	30.62
35.1–36	102	19.77
> =36	90	17.44
Peri-natal asphyxia diagnosed at birth		
Yes	137	26.55
No	379	73.45
Newborn diagnosed with respiratory distress		
Yes	142	27.52
No	374	72.48
Hypothermia diagnosed at admission		
Yes	426	82.56
No	90	17.44
Hypoglycemia diagnosed at admission		
Yes	112	21.71
No	404	78.29
Jaundice		
Yes	127	24.61
No	389	75.39
Newborn diagnosed with clinical sepsis		
Yes	401	77.71
No	115	22.29
Neonate received photo therapy		
Yes	143	27.71
No	373	72.29
Neonate received continuous positive airway pressure (nCPAP)		
Yes	287	55.62
No	229	44.38
Newborn received kangaroo mother care		
Yes	68	13.18
No	448	86.82
Newborn heated with radiant warmer		
Yes	371	71.90
No	145	28.10

### Proportion of preterm neonatal death

**Fig. 1:**
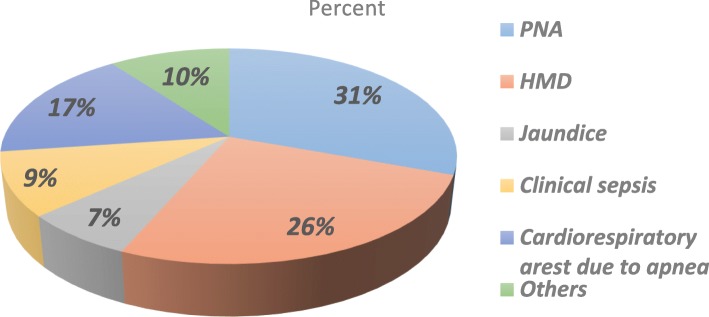
Commonly reported causes of death for preterm neonates admitted in NICU at University of Gondar comprehensive specialized Hospital from January 2016 to March 2018 (*n* = 516)

This finding showed that 149(28.8%) with 95%CI; (25.1, 32.9) neonates were died. Among the deaths 17 (11.4%) died within first 24 h of life and 127(85.23%) were an early neonatal death occurring in the first 7 days of life. The causes of death were multifactorial not single. However, the leading causes were PNA (31%) and HMD (26%) respectively **(**Fig. 1).

### Time to preterm neonate death

The overall median Length of hospital stay for preterm neonates under the study was 7 days, which gave a total of 4527 neonate-days observation and median length of hospital stay were 52 neonate days with an interquartile range of (6, 64) neonate-days.

**Fig. 2:**
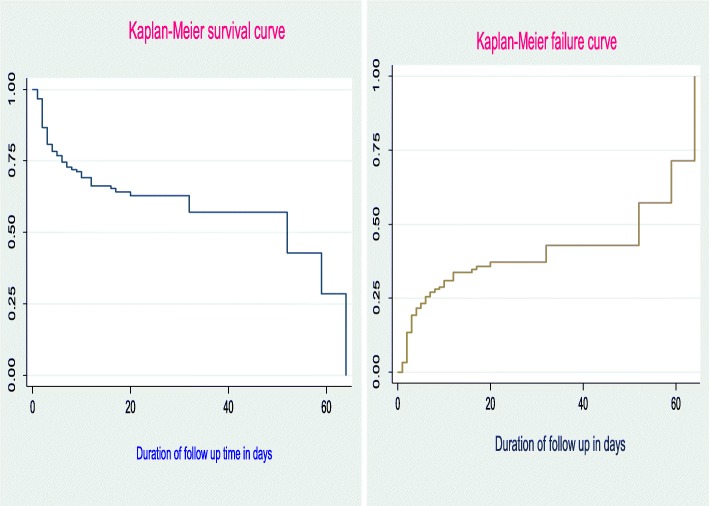
Kaplan-Meier curve of surviving preterm neonates admitted in NICU at University of Gondar comprehensive specialized Hospital from January 2016 to March 2018 (*n* = 516)

The cumulative probability of survival at the end of the first day was 96.71%, at fifth to sixth days was 74.62%, and at 20–32 days was 57.14% (Table 3 and Fig. 2).

**Table 3:** Failure probability of preterm neonates admitted in NICU at University of Gondar comprehensive specialized hospital from January 2016 to March 2018 (*n* = 516)

Time in day	Total at beginning	Deaths	Failure probability %	95% CI
1	516	17	3.29	2.06, 5.25
2	484	50	13.28	10.61,16.57
7	264	60	27.08	23.24, 31.41
14	97	15	33.74	29.04, 38.97
21	42	3	37.15	31.43, 43.55
28	20	0	37.15	31.43, 43.55
63	1	4	100.00

**Fig. 3:**
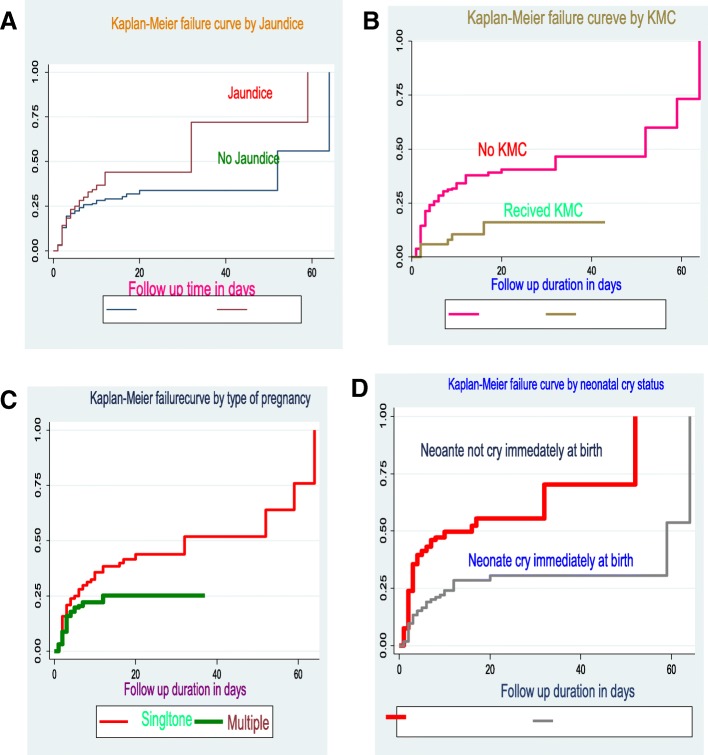
**a-d** Kaplan-Meier curve of failure of preterm neonates admitted in NICU by Jaundice, KMC status type of pregnancy, and neonatal cry status respectively at University of Gondar comprehensive specialized hospital from January 2016 to March 2018 (*n* = 516)

Proportional hazard assumption was assessed using Kaplan Meier survival and Shenfield residual global test and PH assumption was met (chi2 = 4.77 = Prob>chi2 = 0.092) **(**Fig. 3).

### Model comparison criteria

**Fig. 4:**
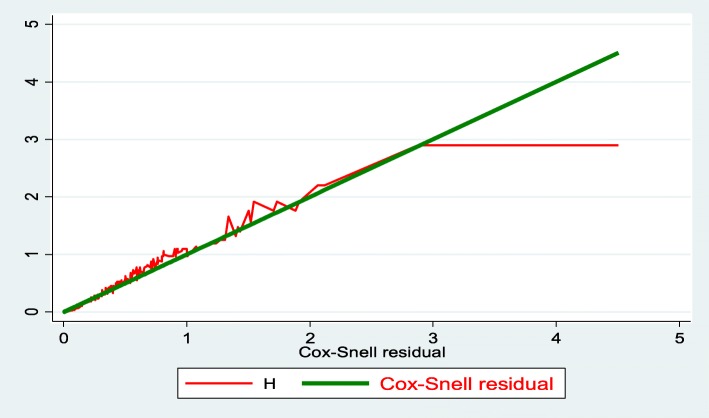
Cox-Snell residual Nelson -Alen cumulative hazard graph on preterm neonates admitted in NICU at University of Gondar comprehensive specialized hospital, Northwest Ethiopia, January 2016 to March 2018 (*n* = 516)

Based on the Akaike Information Criterion, the univariate Gompertz hazard distribution (AIC = 775.85) model was more efficient than Cox-proportional hazard (AIC = 1573.54), parametric exponential model (AIC = 788.63) and Weibull (AIC = 789.97) models (Fig. 4)**.**

### Predictors of time to death for preterm neonates

Univariate and multivariable Gompertz parametric was used for analysis to identify predictors of time to death for preterm neonates from admission to discharge/death in the neonatal intensive care unit.

Findings from bivariate analysis showed that residence of mother, place of delivery type of pregnancy, bad obstetric history, neonatal respiratory distress at admission, clinically diagnosed sepsis, newborns birth weight less than 2000 g, pregnancy complications during index pregnancy, cause of onset of labour, gestational age, weight for gestational age, neonate cried immediately at birth, HMD, PNA, jaundice, receiving KMC, hypoglycemia, hypothermia and maternal illness were significantly associated with time to death of preterm neonates.

However, in the multi-variable analysis place of delivery, type of pregnancy, gestational age, weight for gestational age, neonate cried immediately at birth, HMD, PNA, jaundice, receiving KMC and hypoglycemia were remained statistically significant predictors of time to death of preterm neonates.

The hazard of death among preterm neonates who delivered at home was 2.3 times higher as compared to those who were delivered in the hospital (AHR = 2.29; 95%CI (1.05, 4.98)). The risk of death for a preterm neonate diagnosed with HMD was 3 times as compared to its counterparts (AHR =3.02; 95% CI: (1.86, 4.88)).

As the gestational age increases in 1 week the death rate of preterm neonates was decreased by 18% (AHR = 0.82; 95% CI: (0.74, 0.91)).

The hazard of death for a preterm neonate who cried immediately at birth decreased by 43% as compared to its counterpart (AHR = 0.57; 95% CI: (0.39, 0.83)).

Providing kangaroo mother care for all preterm neonates reduce the risk of death by 77% as compared to not provided kangaroo mother care (AHR = 0.23; 95% CI: (0.10, 0.51)). (Table 4).

**Table 4:** Gompertz hazard model for predictors of time to death among preterm neonates admitted in NICU at University of Gondar specialized referral Hospital from January 2016 to March 2018 (*n = 516*)

Predictor Variables	CHR (95% CI)	AHR (95% CI)
Residence of the mother		
Gondar Town	1	1
Out of Gondar Town	1.51 (1.05, 2.19)	1.25 (0.84, 1.86)
Place of delivery		
Home	2.14 (1.08, 4.23)	2.29 (1.05, 4.98)^*^
Health center	1.30 (0.89, 1.90)	1.09 (0.71, 1.68)
Hospital	1	1
Maternal illness /diseases		
No		1
Yes	1.71 (1.21, 2.40)	1.57 (1.10, 2.26)^*^
Complication during index pregnancy		
No	1	1
Yes	1.55 (1.10, 2.18)	1.26 (0.87, 1.83)
Previous bad obstetrics history		
No	1	1
Yes	1.51 (1.02, 2.24)	1.39 (0.91, 2.12)
Type of pregnancy		
Singleton	1.71 (1.18, 2.46)	2.35 (1.58, 3.50)^**^
Multiple	1	1
Gestational age	0.76 (0.70, 0.82)	0.82 (0.74, 0.91)^**^
Weight for gestational age at birth		
Small	1.56 (1.10, 2.21)	1.65 (1.12, 2.43)^*^
Appropriate	1	1
Neonate cry immediately at birth		
No	1	1
Yes	0.41 (0.30, 0.57)	0.57 (0.39, 0.83)^*^
Neonates diagnosed for HMD		
No	1	1
Yes	4.38 (2.85, 6.74)	3.02 (1.86, 4.88)^**^
Neonatal hypothermia at admission		
No	1	1
Yes	1.68 (1.00, 2.82)	1.18 (0.63, 2.21)
Neonatal respiratory distress at admission		
No	1	1
Yes	1.96 (1.41, 2.71)	1.33 (0.93, 1.90)
Clinically diagnosed PNA		
No	1	1
Yes	2.18 (1.58 3.03)	1.55 (1.09, 2.20)^*^
Clinically diagnosed neonatal sepsis		
No	1	1
Yes	1.68 (1.04, 2.72)	1.46 (0.88, 2.42)
Neonate diagnosed with jaundice		
No	1	1
Yes	1.35 (0.96, 1.90)	1.62 (1.12, 2.35)^*^
Neonate received KMC		
No	1	1
Yes	0.25 (0.12, 0.53)	0.23 (0.10, 0.51)^**^
Neonate diagnosed with hypoglycemia		
No	1	1
Yes	2.10 (1.49, 2.95)	1.75 (1.21, 2.54)^*^
Newborns birth weight in grams		
< 2000	1.36 (0.95, 1.96)	0.64 (0.41, 1.00)
> =2000	1	1
Neonatal temperature measured within 1 h of admission in ^0^C	0.79 (0.71, 0.89)	0.95 (0.82, 1.08)

NB: * *P*-value < 0.05, ** *P*-value < 0.001

## Discussion

In this study, the proportion of preterm neonatal death was 28.8%. The causes of death weren’t single problem rather combination of problems lead to death and the major once were PNA, HMD, jaundice, clinical sepsis and cardiorespiratory arrest due to apnea. Place of delivery, type of pregnancy, maternal illness/diseases, gestational age at birth, weight for gestational age at birth, neonate cry immediately at birth, HMD, PNA, Jaundice, hypoglycemia, kangaroo mother care were found to be predictors of death in this study.

The proportion of death among preterm neonates admitted in University of Gondar comprehensive specialized hospital neonatal intensive care unit was 28.8% (95%CI; (25.1, 32.9)). This finding is in line with studies conducted in a multi-country level analysis reported by WHO and UNICEF 29.3%, and in Kenya 29.6%. However, this finding was higher than studies conducted in a multi-country analysis involving 144 countries by the lead of save the children 15% Cameroon 15.7% Jimma Ethiopia 18.2% and northern rural Ethiopia 23.7%.

This might be due to the difference in the study setting which is entirely admissions from the referral hospital and the study population was focused on only preterm neonates’ most vulnerable groups.

In contrast, this finding was lower than studies conducted in population-based study from low to middle-income countries 37.5%, in urban Pakistan 34% [5], in Jordan 40%, in Johannesburg South Africa 64%, in Tigray region Northern Ethiopia 34%(25–27)and in Jimma University Specialized Hospital, Jimma Ethiopia 34.9%.

This might be due to the fact that with time, even if it is not satisfactory neonatal mortality is decreasing, the skilled birth attendant was increased, NICU is expanded in a well-organized manner, the health seeking and utilization behavior of the community are improved and accessibility of trained health care providers are comparatively increased.

In this study 11.4% of neonatal deaths were within the first 24 h and 85.23% were within the first 7 days known as early neonatal death. This finding was in line with the findings reported by UNICEF [3], a study conducted in Butajira, Ethiopia [6] and a study conducted in Tigray region, Northern Ethiopia.

In this study, the risk of death for preterm neonates delivered at home was 2.3 times higher than those delivered at a hospital (AHR = 2.29, 95%CI (1.05, 4.98). This finding was supported by a study conducted in Jimma Zone in Johannesburg Central Hospital. However, the findings from northern Ethiopia, Kiltie Awlaelo Health and Demographic Surveillance System and Ethiopia Demographic and Health Surveys (DHS) data contradicted this finding [14]. This might be due to the difference in study population where the current finding was entirely on preterm neonates only as compared to those included both preterm and term neonates. The other difference was study design which was an institution based on hospital admissions compared with community-based surveys in the presence of time variation affecting the community health-seeking behavior and level of awareness.

This finding showed that a neonate delivered from mothers who had illness/disease increased the risk of death by 53% as compared with their counterpart (AHR = 1.53, 95%CI (1.07, 2.21)). This finding was supported with a study conducted in northern Ethiopia, in Ethiopia, DHS data, in the sub-urban hospitals of Cameroon and Johannesburg Central Hospital. This similarity might be due to the direct effect of maternal diseases (HIV, Malaria, Pyelonephritis and other febrile diseases) affecting the pregnancy to result in preterm labour and acquired infections leading to preterm neonatal death.

Being singleton pregnancy in this finding was 2.18 times higher risk for death of preterm neonates as compared to multiple pregnancies (AHR = 2.18 (95%CI 1.47, 3.25)). This find was in contrary to the findings conducted in Jimma Zone, in northern Ethiopia and in Ethiopia, DHS data. This might be because of mothers with known multiple pregnancies had better prenatal care and delivery care. In addition study population difference that the current study focuses only on preterm who had maturity problems. From those preterm neonates, multiple pregnancies mature earlier than singleton once which is clinically sound justification. But other studies were account for all neonates.

In this study, as the gestational age increase in a week the risk of death was decreased by 18% (AHR of 0.82; (95%CI 0.74, 0.91)). This finding was in line with a study conducted in Jimma University specialized hospital and in Addis Ababa St Paul’s Hospital Millennium Medical College. This was supported by the clinical evidence that as gestational age increases fetal maturity will be maximized and risk of developing different life-threatening complications associated with prematurity may decrease and risk of death will be reduced.

This study showed that a neonate who was small for gestational age at birth was 1.7 times at higher risk of death compared to those who were appropriate for gestational age (AHR = 1.72, 95%CI (1.17, 2.53)). This was supported by a study conducted in Jimma Zone in Ethiopia, DHS data, in Addis Ababa St Paul’s Hospital Millennium Medical College and Johannesburg Central Hospital. The possible reasons might be due to that if small for gestational age the occurrence of life-threatening complications like hypoglycemia and hypothermia which lead to death is high compared to appropriate for gestational age neonates.

The risk of death was reduced by 42% for a neonate cried immediately at birth (indicating that the neonate has good APGAR) as compared to the counterpart with (AHR = 0.58, 95%CI (0.40, 0.84)). This result was comparable with the findings conducted in Addis Ababa St Paul’s Hospital Millennium Medical College indirectly with study conducted in sub-urban hospital of Cameroon, Johannesburg Central Hospital and Taubaté University Hospital, Brazil [13].

In the current study the risk of preterm neonatal death among cases of HMD was 3.2 times higher compared to none cases (AHR = 3.21, 95%CI (1.96, 5.25)). This finding was comparable with findings conducted in Jimma University specialized hospital and in Johannesburg Central Hospital. This might be due to HMD is disease of prematurity affecting respiratory function which is vital for survival and primary cause of death in preterm neonates.

PNA increases the risk of death by 52%, (AHR = 1.52, 95%CI (1.06, 2.16)). This finding was similar with a study conducted in Jimma University specialized hospital and in Addis Ababa St Paul’s Hospital Millennium Medical College. The possible reasons might be that PNA is one of the leading causes of neonatal death where the quality and access of emergency obstetric newborn and comprehensive emergency obstetric services are inadequate in every clinical setting of the country.

Preterm neonates diagnosed with jaundice had 1.65 times higher risk of death than their counter parts (AHR = 1.65, 95%CI (1.14, 2.41)). This finding was in line with a study conducted in Jimma University specialized hospital. This might be due to that preterm neonates are at risk of developing jaundice due to gastrointestinal immaturity, liver enzyme deficiency leading to excess production of bilirubin to result in brain toxicity and death.

Hypoglycemia was significantly associated with the risk of death for preterm neonates, (AHR = 1.57, 95%CI (1.08, 2.29)). This finding is supported by the clinical practice that preterm neonates are highly affected and lead to death due to hypoglycemia because of luck of adipose fat tissue which serves as the source of glucose to adapt the extrauterine life until they maintain through feeding.

This finding showed that the risk of death among preterm neonates received kangaroo mother care was lowered by 73% (AHR = 0.25, 95%CI (0.13, 0.58)). This finding is supported by the clinical practice that kangaroo mother care is recommended for all preterm neonates until they reach term or show signs of winning the care to prevent hypothermia by reducing body surface area to the external environment. And also reduce the risk of hypoglycemia by easily accessing breastfeed as demand day and night.

## Conclusions

In this study the proportion of preterm neonatal death was high. PNA, HMD, jaundice, clinical sepsis and cardiorespiratory arrest due to apnea were the leading causes of death. Place of delivery, type of pregnancy, maternal illness/diseases, weight for gestational age at birth, HMD, PNA, Jaundice, hypoglycemia were significant risk factors for time to death. On the other way gestational age, neonate cry immediately at birth, kangaroo mother care were found to be preventive predictors for time to death in this study. All responsible bodies should work on quality care at ANC to maximize maternal health conditions, access NICU with infrastructures and skilled manpower at health institutions and give special care for preterm to avoid complications due to preterm.

## Abbreviations

ANC: Antenatal care
AOR: Adjusted odds ratio
APGAR: Appearance pulse grimace activity respiration
APH: Antepartum hemorrhage
BSc: Bachelors of science
CI: Confidence interval
COR: Crude odd ratio
EDHS: Ethiopian demographic and health survey
GA: Gestational age
HIV: Human immune-deficiency virus
HMD: Hyaline membrane disease
HR: Hazard ratio
IRB: Institutional review board
KMC: Kangaroo mother care
NCPAP: Nasal continuous positive air pressure
NEC: Necrotizing enterocolitis
NGOs: Non-governmental organizations
NICU: Neonatal intensive care unit
NMR: Neonatal mortality rate
OR: Odds ratio
PIH: Pregnancy induced hypertension
PNA: Perinatal asphyxia
PROM: Prelabour rupture of membrane
PT: Preterm
RDS: Respiratory distress syndrome
UN: United Nations
UNICEF: United Nations international emergency children fund
WHO: World health organization
